# The Optimal Strategy of Dual Antiplatelet Therapy after Percutaneous Coronary Intervention with Drug-Eluting Stent

**DOI:** 10.3390/jcm11154465

**Published:** 2022-07-31

**Authors:** Mengjin Hu, Xiaojin Gao, Jingang Yang, Yuejin Yang

**Affiliations:** State Key Laboratory of Cardiovascular Disease, Fuwai Hospital, National Center for Cardiovascular Diseases, Chinese Academy of Medical Sciences & Peking Union Medical College, Beijing 100037, China; 18393911603@163.com (M.H.); yangjingang@fuwai.com (J.Y.)

**Keywords:** percutaneous coronary intervention, drug-eluting stents, dual antiplatelet therapy, P2Y12 inhibitor

## Abstract

Objective: To test the optimal strategy of dual antiplatelet therapy (DAPT) after implantation of drug-eluting stents (DESs) according to specific DAPT time and subsequent monotherapy. Methods: We searched the Cochrane Central Register of Controlled Trials (CENTRAL), Medline, Embase, and Web of Science to identify randomized controlled trials (RCTs). Six DAPT strategies were compared: 1-month DAPT followed by P2Y12 inhibitor monotherapy, 3-month DAPT followed by P2Y12 inhibitor monotherapy, 3-month DAPT followed by aspirin monotherapy, 6-month DAPT followed by aspirin monotherapy, 12-month DAPT, and >12-month DAPT. Pooled odd ratios (ORs) with 95% credible intervals (CrIs) were calculated to summarize the effect of each strategy tested. Results: We identified 24 RCTs containing 81,405 patients. In comparison with 12-month DAPT, 3-month DAPT followed by P2Y12 inhibitor monotherapy reduced net clinical events (OR: 0.72; CrI: 0.55–0.94). Major bleeding (OR: 0.57; CrI: 0.34–1.00) was marginally decreased without impact on ischemic events (OR: 0.93; CrI: 0.68–1.29). Moreover, the benefits of 3-month DAPT (P2Y12 inhibitor) were consistent for male patients with acute coronary disease, young age, complex lesion, single-vessel disease, low body mass index, and without diabetes. Although >12-month DAPT was associated with a lower risk of myocardial infarction (OR: 0.67; CrI: 0.51–0.93), the risk of major bleeding (OR: 1.70; CrI: 1.10–2.70) was increased. Conclusion: Among patients treated with DESs, 3-month DAPT followed by P2Y12 inhibitor monotherapy may be the optimal antiplatelet strategy, while DAPT beyond 1 year reduces myocardial infarction at the expense of increased major bleeding.

## 1. Introduction

Drug-eluting stent (DES) implantation during percutaneous coronary intervention (PCI) has resulted in improved clinical outcomes and has been widely used in patients with coronary artery disease [1]. After PCI, patients are commonly prescribed dual antiplatelet therapy (DAPT), which is the combination of aspirin and a platelet P2Y12 inhibitor. Although DAPT remains the cornerstone of pharmacological treatment aimed at preventing atherothrombotic complications in patients with various coronary artery disease manifestations [2], there is concern that DESs may be associated with an increased propensity for late and very late stent thrombosis, a relatively rare but life-threatening event [3]. The observed high rate of stent thrombosis with DESs motivated the interventional community to extend DAPT beyond 12 months [4]. However, prolonged DAPT may increase major bleeding rates compared with treatment with aspirin alone [5]. Worse still, the higher incidence of bleeding episodes is strongly associated with all-cause death [6]. Therefore, the optimal or minimal necessary duration of DAPT and the risk-benefit ratio for long-term DAPT remain undetermined for patients receiving DESs. In recent years, a number of randomized trials have been published to examine the optimal duration of DAPT after DES implantation, ranging from 1 month to as long as 48 months [7,8], yet no consensus has been arrived on the optimal duration of DAPT. Moreover, except for the traditional continuation of aspirin, discontinuing aspirin while maintaining administration of a P2Y12 inhibitor monotherapy after DAPT was also introduced to reduce the risk of bleeding [9,10,11]. Therefore, after DAPT, which antiplatelet agent (aspirin or P2Y12 inhibitor) should be continued is still uncertain.

Currently, six DAPT strategies are available: 1-month DAPT followed by P2Y12 inhibitor monotherapy, 3-month DAPT followed by P2Y12 inhibitor monotherapy, 3-month DAPT followed by aspirin monotherapy, 6-month DAPT followed by aspirin monotherapy, 12-month DAPT, and >12-month DAPT. Although numerous meta-analyses have been published in the DAPT duration domain [12,13,14,15], no study has been published to compare the effects of all available modalities within a single analytical framework, especially to distinguish P2Y12 inhibitor monotherapy and aspirin monotherapy. The newly published 2021 American College of Cardiology (ACC)/American Heart Association classification (AHA) guidelines also highlight that “no trial has compared short-term DAPT followed by P2Y12 monotherapy with short-term DAPT followed by aspirin alone” [16]. Therefore, in this network meta-analysis, we sought to determine the most effective DAPT strategy in patients who had undergone initial PCI with the placement of DESs according to more specific DAPT time and subsequent monotherapy.

## 2. Methods

This report complies with the PRISMA (preferred reporting items for systematic reviews and meta-analyses) network meta-analysis extension statement [17]. Since the summary data were obtained from published randomized trials with approval from respective institutional review committees, no further sanction was required for our network meta-analysis. This meta-analysis was registered at the PROSPERO international prospective register of systematic reviews (CRD42021291447).

### 2.1. Search Strategy and Eligibility Criteria

We conducted a systematic search of the published full-text literature from the databases Cochrane Central Register of Controlled Trials (CENTRAL), Medline, Embase, and Web of Science on 8 March 2022. The medical subject headings or keywords included the following: dual antiplatelet therapy, DAPT, P2Y12 inhibitor, clopidogrel, ticagrelor, prasugrel, aspirin, drug-eluting stent, DESs, percutaneous coronary intervention, PCI, randomized controlled trial, randomized trial, trial. Moreover, relevant randomized trials from reference lists of identified systematic reviews, meta-analyses, and relevant reviews were additionally hand-searched to supplement the search of the electronic databases. Randomized trials that compared different durations of DAPT and reported clinical outcomes after DES implantation were included.

### 2.2. Outcomes

The primary outcomes were net clinical events (combination of one or more ischemic events and bleeding), ischemic events (any combination of all-cause death, cardiac death, myocardial infarction, stroke, definite or probable stent thrombosis, revascularization), myocardial infarction, and major bleeding. The secondary outcomes were all-cause death, cardiac death, stroke, definite or probable stent thrombosis, target vessel/lesion revascularization (TVR/TLR), revascularization, and any bleeding.

### 2.3. Statistical Analysis

Bayesian network meta-analysis was performed with a random-effect model, and odd ratios (ORs) with 95% credible interval (CrIs) were calculated for all outcomes of interest. To rank the DAPT strategies for each outcome, the surface under the cumulative ranking curve (SUCRA) was used. SUCRA values vary between 0 and 100%: the higher the value, the higher the likelihood that a strategy is in the top rank or highly effective [18]. Six Markov chains were run simultaneously with 30,000 simulated draws after a burn-in of 10,000 iterations. We statistically evaluated consistency by separating out direct evidence from indirect evidence using node splitting. The risk of bias for all included randomized trials was assessed using the Cochrane risk of bias assessment tool [19], and publication bias was investigated with comparison-adjusted funnel plots.

Subgroup analyses of primary outcomes according to individual trials were performed as follows: acute coronary syndrome (ACS) and stable angina, young patients and old patients, diabetes and no diabetes, prasugrel/ticagrelor and clopidogrel, first-generation DESs and new-generation DESs, Caucasian and non-Caucasian, complex lesion (type B2 or C lesions according to the modified ACC/AHA criteria), single-vessel disease and multivessel disease, male and female, heart failure and no heart failure, renal failure and no renal failure, single stent and multistent, low body mass index (BMI), and high BMI. All analyses were conducted using R software (version 3.4.3) equipped with the “gemtc” package.

## 3. Results

After examining a total of 4732 abstracts, we retrieved 55 full-text papers for further consideration. The PRISMA flowchart describing the inclusion process, including the reasons for exclusion, is presented in Supplementary Figure S1. In brief, 24 randomized trials with 81,405 patients met the inclusion criteria. Two studies were comparisons between 12-month DAPT and 1-month DAPT followed by P2Y12 inhibitors [7,20], three studies were comparisons between 12-month DAPT and 3-month DAPT followed by P2Y12 inhibitors [9,10,11], three studies were comparisons between 12-month DAPT and 3-month DAPT followed by aspirin [21,22,23], seven studies were comparisons between 12-month DAPT and 6-month DAPT followed by aspirin [24,25,26,27,28,29,30], four studies were comparisons between >12-month DAPT and 6-month DAPT followed by aspirin [31,32,33,34], and five studies were comparisons between >12-month DAPT and 12-month DAPT [8,35,36,37,38]. The total number of patients in the 1-month DAPT (P2Y12 inhibitor), 3-month DAPT (P2Y12 inhibitor), 3-month DAPT (aspirin), 6-month DAPT (aspirin), 12-month DAPT, and >12-month DAPT groups was 9480, 6577, 3373, 11,045, 35,743, and 15,187, respectively. The baseline characteristics of included randomized trials are shown in Supplementary Table S1. With regard to the risk of bias, most of the studies were in the low categories for risk of bias, except for the high risk of performance bias (Supplementary Figure S2).

**Figure 1:**
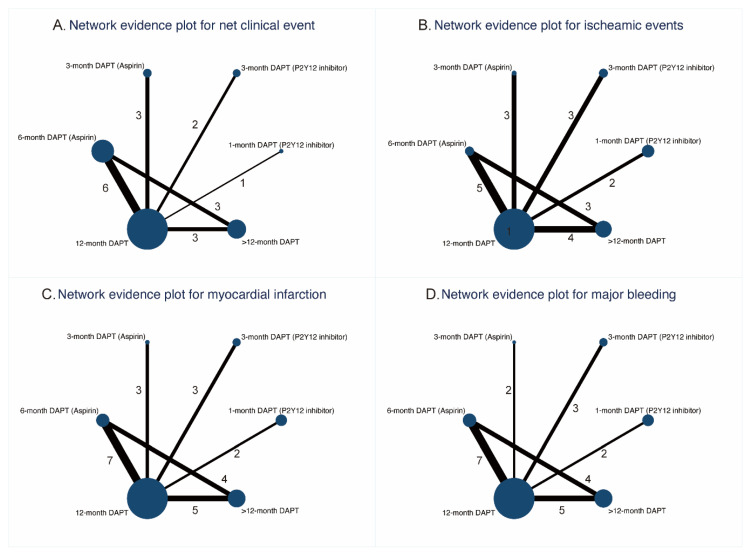
Network evidence plots for primary outcomes.

The network plots for primary and secondary outcomes are shown in Figure 1 and Supplementary Figure S3, respectively. The nodes and edges are weighted according to the number of available treatment formats and comparisons. Visual analysis of funnel plots demonstrated no evidence of publication bias (Supplementary Figure S4).

### 3.1. Primary Outcomes

#### 3.1.1. Net Clinical Events

**Figure 2:**
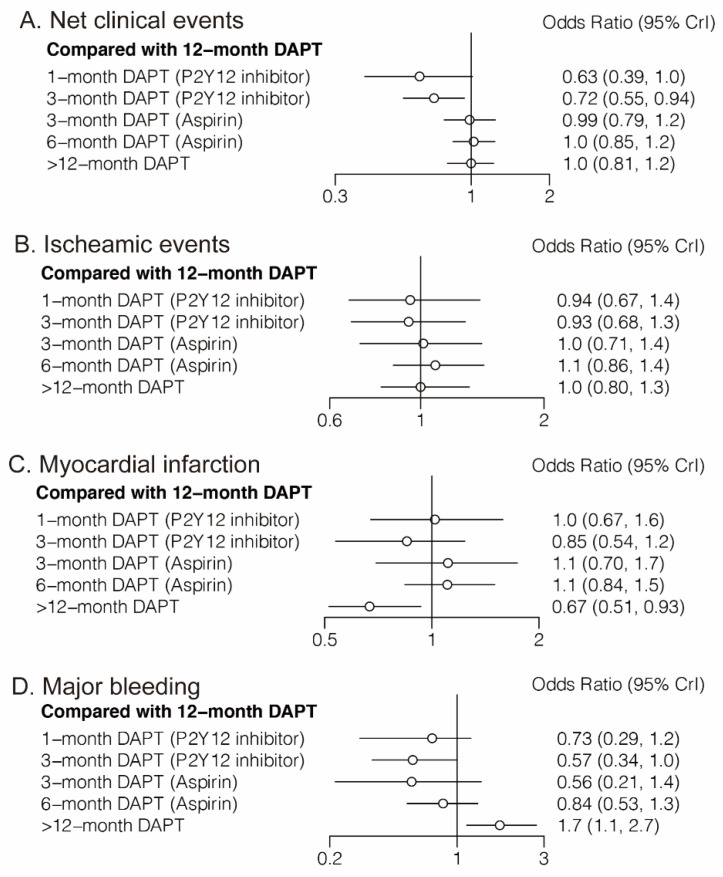
Network Meta-Analysis for Primary Outcomes.

**Figure 3:**
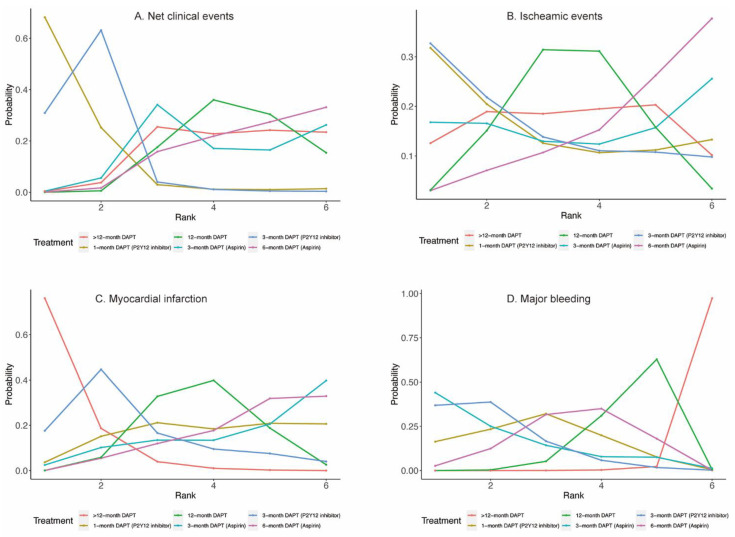
Rankograms for Primary Outcomes.

Eighteen randomized trials (42,253 patients) reported 1868 (4.42%) net clinical events. In comparison with 12-month DAPT, 3-month DAPT followed by P2Y12 inhibitor monotherapy significantly reduced net clinical events (OR: 0.72; CrI: 0.55–0.94). A trend towards decreased risk of net clinical events was also observed with 1-month DAPT (P2Y12 inhibitor) (Figure 2A). However, 6-month DAPT (aspirin) was associated with a high risk of net clinical events compared with 3-month DAPT (P2Y12 inhibitor) (OR: 1.42; CrI: 1.03–1.98) (Table 1). Figure 3A demonstrated that 1-month DAPT (P2Y12 inhibitor) and 3-month DAPT (P2Y12 inhibitor) were highly effective in reducing net clinical events.

#### 3.1.2. Ischemic Events

Twenty randomized trials (71,248 patients) reported 3139 (4.41%) ischemic events. No significant differences were observed between any comparison of DAPT strategies (Figure 2B and Table 1).

#### 3.1.3. Myocardial Infarction

Twenty-four randomized trials (81,339 patients) reported 1703 (2.09%) myocardial infarction events. Compared with 12-month DAPT, >12-month DAPT was associated with a lower risk of myocardial infarction (OR: 0.67; CrI: 0.51–0.93) (Figure 2C). The risk of myocardial infarction was also lower with >12-month DAPT compared with 6-month DAPT (aspirin) (OR: 0.61; CrI: 0.44–0.86) (Table 1). As expected, >12-month DAPT ranked first in decreasing the risk of myocardial infarction (Figure 3C).

#### 3.1.4. Major Bleeding

Twenty-three randomized trials (79,879 patients) reported 1019 (1.28%) major bleeding events. One-month DAPT (P2Y12 inhibitor), 3-month DAPT (P2Y12 inhibitor), 3-month DAPT (aspirin), 6-month DAPT (aspirin), and 12-month DAPT were all associated with reduced risk of major bleeding compared with >12-month DAPT. Moreover, a trend towards decreased risk of major bleeding was also observed with 3-month DAPT (P2Y12 inhibitor) compared with 12-month DAPT (Table 1). Longer than 12-month DAPT was least effective in reducing the risk of major bleeding (Figure 3D).

### 3.2. Secondary Outcomes

#### 3.2.1. All-Cause Death and Cardiac Death

**Figure 4:**
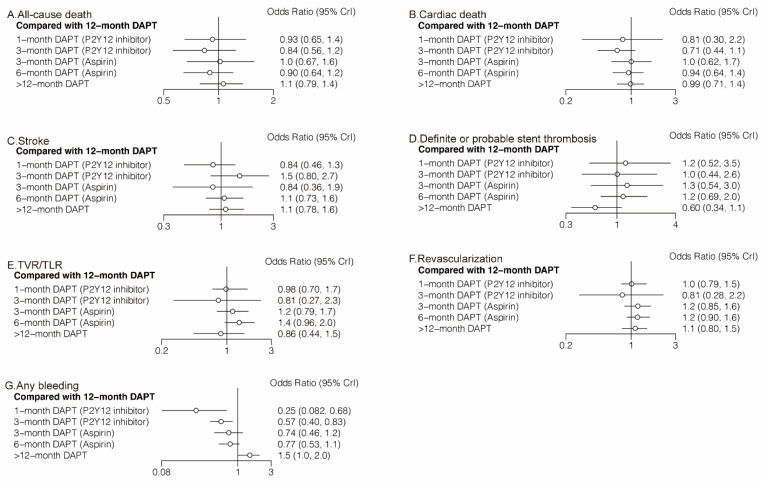
Network Meta-Analysis for Secondary Outcomes.

**Figure 5:**
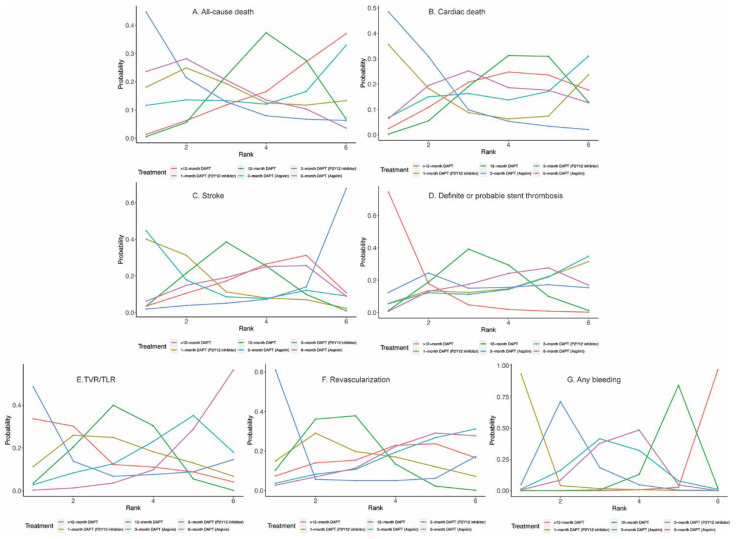
Rankograms for Secondary Outcomes.

Twenty-four (81,339 patients) and twenty (57,411 patients) randomized trials reported 1530 (1.88%) all-cause deaths and 593 (1.03%) cardiac deaths, respectively. No significant differences were observed between any comparison of DAPT strategies (Figure 4A,B, Table 2) in all-cause death or cardiac death. However, 3-month DAPT (P2Y12 inhibitor) ranked first in decreasing the risks of all-cause death (Figure 5A) and cardiac death (Figure 5B).

#### 3.2.2. Stroke and Definite or Probable Stent Thrombosis

Twenty-four (81,339 patients) randomized trials reported 610 (0.75%) stroke events. No significant differences were observed between any comparison of DAPT strategies (Figure 4C, Table 2). Twenty-four (81,339 patients) randomized trials reported 492 (0.60%) definite or probable stent thrombosis events. Six-month DAPT (aspirin) was associated with a higher risk of definite or probable stent thrombosis compared with >12-month DAPT (OR: 1.92; CrI: 1.03–3.71) (Table 2), and >12-month DAPT ranked first in decreasing definite or probable stent thrombosis (Figure 5D).

#### 3.2.3. TVR/TLR and Revascularization

Fourteen (40,272 patients) and seventeen (49,277 patients) randomized trials reported 1444 (3.59%) TVR/TLR and 2625 (5.33%) revascularization events, respectively. There were no significant differences between any comparison of DAPT strategies in TVR/TLR or revascularization (Table 2). However, 3-month DAPT (P2Y12 inhibitor) ranked first in decreasing the risks of TVR/TLR (Figure 5E) and revascularization (Figure 5F).

#### 3.2.4. Any Bleeding

Eighteen (51,551) randomized trials reported 1631 (3.16%) (any) bleeding events. Six-month DAPT (aspirin) significantly increased the risk of any bleeding compared with 1-month DAPT (P2Y12 inhibitor) (OR: 3.09; CrI: 1.07–9.82), 12-month DAPT significantly increased the risk of any bleeding compared with both 1-month DAPT (P2Y12 inhibitor) (OR: 3.99; CrI: 1.46–12.23) and 3-month DAPT (P2Y12 inhibitor) (OR: 1.74; CrI: 1.20–2.49), and >12-month DAPT significantly increased the risk of any bleeding compared with any DAPT strategies (Table 2). 

**Figure 6:**
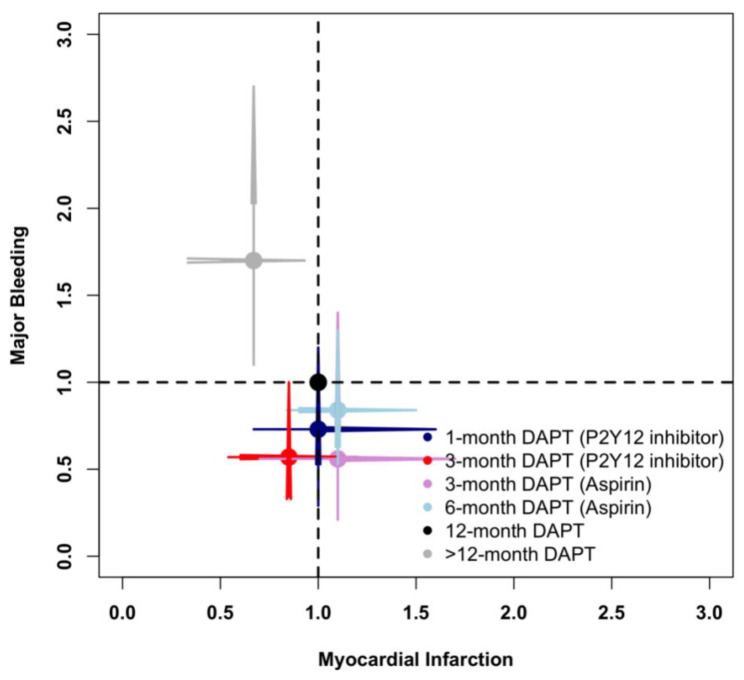
Comparison of Myocardial Infarction and Major Bleeding in the Network Meta-Analysis.

Figure 6 illustrates the risks of myocardial infarction and major bleeding of different DAPT strategies compared with standard 12-month DAPT. Overall, >12-month DAPT decreased myocardial infarction at the expense of increased major bleeding, whereas 3-month DAPT (P2Y12 inhibitor) decreased major bleeding without increasing myocardial infarction events.

### 3.3. Network Coherence

The network node-split outcomes for primary and secondary outcomes revealed that there were no noticeable differences between direct and indirect estimates in closed loops that allowed the assessment of network coherence (Supplementary Figure S5).

### 3.4. Subgroup Analyses of Primary Outcomes

**Figure 7:**
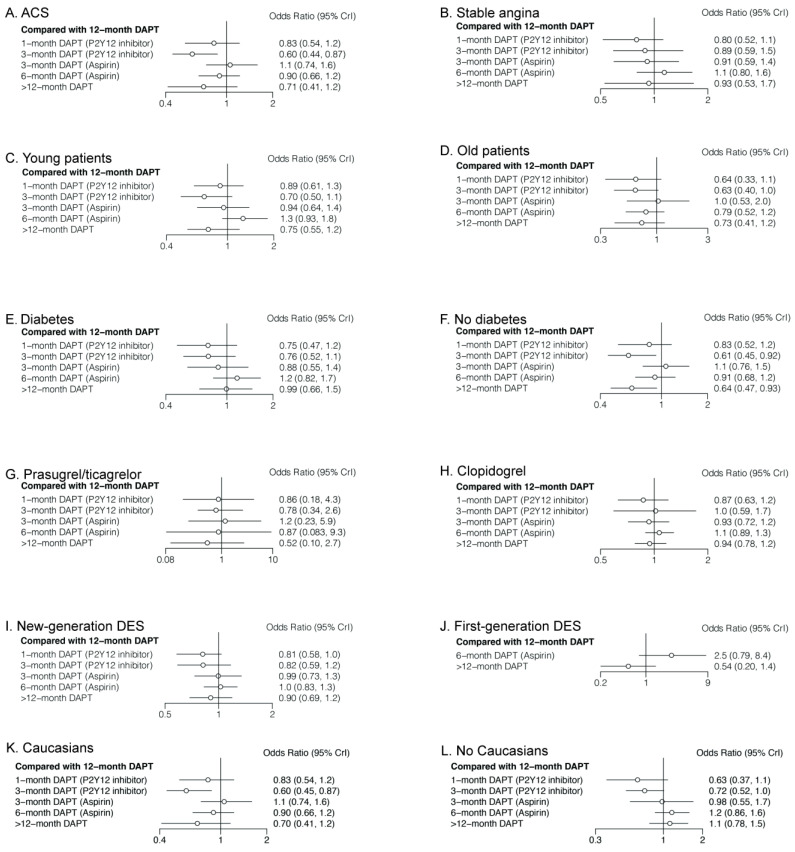
Comparision of Primary Outcomes in Different Patients.

As shown in Table 3 and Figure 7, in patients with ACS, both 3-month DAPT (aspirin) (OR: 1.76; CrI: 1.05–2.87) and 12-month DAPT (OR: 1.67; CrI: 1.15–2.25) significantly increased primary outcomes compared with 3-month DAPT (P2Y12 inhibitor). However, no significant differences were observed in patients with stable angina. In young patients, 6-month DAPT (aspirin) was associated with a higher risk of primary outcome compared with 3-month DAPT (P2Y12 inhibitor) (OR: 1.78; CrI: 1.09–2.93), whereas >12-month DAPT significantly reduced primary outcomes compared with 6-month DAPT (aspirin) (OR: 1.78; CrI: 1.09–2.93). No significant difference was observed in old patients or patients with diabetes. However, in patients without diabetes, 3-month DAPT (P2Y12 inhibitor) was associated with a lower risk of primary outcomes compared with both 3-month DAPT (aspirin) (OR: 0.57; CrI: 0.37–0.98) and 12-month DAPT (OR: 0.61; CrI: 0.45–0.92). There were no significant differences in primary outcomes between any comparison of DAPT strategies in patients receiving prasugrel/ticagrelor, clopidogrel, or new-generation DESs. In patients receiving first-generation DESs, 6-month DAPT (aspirin) significantly increased primary outcomes compared with >12-month DAPT (OR: 4.63; CrI: 1.05–21.99). Three-month DAPT (P2Y12 inhibitor) was consistently beneficial, regardless of population origin. 

Subgroup analyses of primary outcomes with five DAPT strategies are shown in Supplementary Table S2 and Supplementary Figure S6, and subgroup analyses with four DAPT strategies are shown in Supplementary Table S3 and Supplementary Figure S7. In brief, for male patients with complex lesions, single vessel disease, or low BMI, 3-month DAPT (P2Y12 inhibitor) was associated with a lower risk of primary outcome. However, there were no significant differences in female patients with multivessel disease or high BMI. Heart failure, renal failure, or the number of stents had no impact on clinical outcomes either.

## 4. Discussion

The optimal duration of DAPT is an important clinical issue, given the large number of patients treated with DESs, the costs and bleeding risks related to DAPT, the potentially life-threatening consequences of stent thrombosis, and the potential benefits of DAPT in preventing ischemic events. In this network meta-analysis based on 24 randomized trials and 81,405 patients, we found that 3-month DAPT followed by P2Y12 inhibitor monotherapy significantly reduced net clinical events, which was mainly due to reduced risks of major and any bleeding, without increasing the incidence of composite or separate ischemic events Moreover, the benefits of 3-month DAPT (P2Y12 inhibitor) were more evident for male patients with ACS, young age, complex lesion, single-vessel disease, low BMI, and without diabetes. Although longer DAPT (>12 months) significantly reduced myocardial infarction, the risk of major bleeding was also increased.

Aspirin has proven benefits and has become the cornerstone for antiplatelet therapy in secondary prevention of cardiovascular disease. Recently, discontinuing aspirin while maintaining administration of a P2Y12 inhibitor after DAPT was introduced to reduce the risk of bleeding [9,10,11]. In our network meta-analysis, 3-month DAPT followed by P2Y12 inhibitor monotherapy was associated with a better prognosis. P2Y12 inhibitor monotherapy after DAPT might limit bleeding risk while retaining the ischemic benefits associated with prolonged DAPT, and continued long-term P2Y12 inhibitor monotherapy may provide greater ischemic protection than aspirin alone [39]. A previous meta-analysis demonstrated that short duration of DAPT followed by aspirin monotherapy was associated with an increased risk of myocardial infarction and stent thrombosis, while prolonged DAPT increased the risk of bleeding, which may offset the benefit derived from reduced ischemic events [40]. Therefore, neither prolonged DAPT nor short duration of DAPT followed by aspirin monotherapy is fully satisfactory. However, the newly developed antiplatelet strategy that continues DAPT for 3 months followed by P2Y12 inhibitor monotherapy may maintain efficacy for ischemic events while reducing the bleeding risk after PCI.

The better prognosis associated with 3-month DAPT (P2Y12 inhibitor) could be explained by several plausible reasons. First, it is reported that P2Y12 inhibitor monotherapy inhibited hemostatic system activation to a comparable extent relative to DAPT [41], yet aspirin alone provided little additional inhibition of platelet aggregation in the presence of a P2Y12 inhibitor [42]. At the same time, P2Y12 inhibitor monotherapy is not associated with a higher risk of cerebrovascular events than DAPT [10]. Second, the risk of bleeding was significantly lower with P2Y12 inhibitor monotherapy than with DAPT. Reducing bleeding after PCI is of great significance, as bleeding has a strong association with subsequent all-cause death and major adverse cardiovascular events, approximating or even exceeding that associated with myocardial infarction [6,43]. Third, as observed in the present network meta-analysis, most patients were treated with new-generation DESs. Several studies have reported significant decreases in all-cause death and myocardial infarction with new-generation DESs compared with first-generation DESs. The risk of definite or probable stent thrombosis is also on average 50% lower with new-generation DESs [44,45]. Given the very low rate of stent thrombosis (0.60%) and relatively high rate of major bleeding (1.28%) in the present meta-analysis, reducing major bleeding with short-term DAPT may be more important than attempting further reduction of stent thrombosis with intensive DAPT. In this context, it seems that 3-month DAPT followed by P2Y12 inhibitor monotherapy might be an attractive option after implantation of current-generation DESs, as it is not associated with an increased ischemic events while reducing bleeding events. Similarly to our findings, the newly published 2021 ACC/AHA guidelines recommend that in selected patients receiving PCI, shorter-term DAPT (1–3 months) followed by P2Y12 inhibitor monotherapy is reasonable in reducing the risk of bleeding (Class IIa, Level A) [16]. In our network meta-analysis, we further demonstrated that the benefit was more evident in the 3-month DAPT (P2Y12 inhibitor) group than in the 1-month DAPT (P2Y12 inhibitor) group.

Patients presenting with ACS have a higher risk of recurrent ischemic events than those with stable ischemic heart disease [46]. Therefore, the benefits of prolonged DAPT might be more prominent in patients with ACS than in those with stable ischemic heart disease. The 2016 ACC/AHA guidelines recommend that in patients with stable ischemic heart disease treated with DES implantation, DAPT should be given for at least 6 months (Class I, Level B), while in patients with ACS, DAPT should be given for at least 12 months (Class I, Level B) [47]. Similarly, the 2018 European Society of Cardiology (ESC) and European Association for Cardio-Thoracic Surgery (EACTS) guidelines call for 6 months of DAPT for patients with stable coronary artery disease, regardless of DESs type (Class I, Level A), and 12 months of DAPT for patients with ACS, unless there are contraindications, such as an excessive risk of bleeding (Class I, Level A) [48]. In the DAPT-STEMI trial published in 2018, six (*n* = 432) versus 12 months of DAPT (*n* = 438) were compared after implanting second-generation DESs in patients presenting with ST-elevation myocardial infarction (STEMI). After 2 years of follow-up, the primary end point, defined as a composite of all-cause death, myocardial infarction, revascularization, stroke, and major bleeding, had occurred in 4.8% of patients receiving 6-month DAPT versus 6.6% of patients receiving 12-month DAPT [hazard ratio (HR): 0.73; 95% CI: 0.41–1.27; *p* = 0.26]. Noninferiority was also met [30]. Therefore, it seems that 6-month DAPT was noninferior to the currently recommended regimen of 12-month DAPT, even for STEMI patients after primary PCI with second-generation DESs. Moreover, in most cases, the nonsignificant lesions with traits of “vulnerability” progress through an asymptomatic plaque rupture and heal to more stable atherosclerotic lesions, while other plaques remain unchanged [49]. As such, while DAPT as secondary prevention may reduce cardiovascular events originating from atherosclerosis progression in the coronary tree, these events are rare, and the benefits of reduced ischemic events may not outweigh the increase in risk of bleeding associated with prolonged DAPT. Meanwhile, major bleeding is an adverse event that is strongly related to all-cause death [6], and bleeding is a stronger predictor of non–cardiovascular death than stent thrombosis events [50], Based on this evidence and considering that atherosclerosis is a lifelong disease, shortening the duration of DAPT in secondary prevention may be more advisable.

In the network meta-analysis conducted by Yin et al. [15], 17 randomized trials were included. Compared with short-term DAPT (<6 months), long-term DAPT (>12 months) led to higher rates of major bleeding, and standard DAPT (12 months) was associated with an increased risk of any bleeding. For patients with ACS, short-term DAPT presented similar efficacy and safety with standard-term DAPT. For patients implanted with newer-generation DESs, long-term DAPT resulted in more all-cause deaths than short-term DAPT. Therefore, it seems that short-term DAPT should be taken into consideration for patients receiving DESs. In another network meta-analysis, short-term (<6-month) DAPT followed by aspirin or P2Y12 inhibitor monotherapy, midterm (6-month) DAPT, 12-month DAPT, and extended-term (>12-month) DAPT after PCI with DESs were compared. The results indicated that short-term DAPT followed by P2Y12 inhibitor monotherapy reduced major bleeding, whereas extended-term DAPT reduced myocardial infarction at the expense of more bleeding events [51]. In the present meta-analysis, we included more randomized trials and further divided short-term (<6-month) DAPT followed by P2Y12 inhibitor monotherapy into 1-month DAPT (P2Y12 inhibitor) and 3-month DAPT (P2Y12 inhibitor), and the results indicated that the better prognosis associated with short-term (<6-month) DAPT followed by P2Y12 inhibitor monotherapy was mainly driven by 3-month DAPT followed by P2Y12 inhibitor monotherapy. As far as we are concerned, this is the largest meta-analysis and includes the largest number of randomized trials. Moreover, subgroups were also made to investigate the optimal duration of DAPT according to patients’ characteristics. The subgroup analyses demonstrated consistent benefit associated with 3-month DAPT (P2Y12 inhibitor). However, we cannot exclude the possibility that within-subgroup estimates of treatment effect may be underpowered, due to the limited number of patients. Additionally, given the relatively infrequent occurrence of all-cause death, myocardial infarction, or stent thrombosis, our findings should be confirmed or refuted through larger randomized trials with long-term follow-up. Considering the complexity of disease conditions and difficulty in keeping a balance between ischemic and bleeding events, personalized DAPT had better be taken into consideration at well.

## 5. Limitations

First, as shown in Supplementary Figure S2, most randomized trials were performed in an open-label manner, which could have led to bias. However, the outcome assessment was almost performed in a blind manner. Second, since patients were not randomly assigned to a specific P2Y12 inhibitor drug or stent type, direct comparisons between different stent types or drugs may be confounded. In patients receiving DESs, novel P2Y12 inhibitors (prasugrel and ticagrelor) were associated with significantly reduced rates of ischemic events compared with clopidogrel [52,53]. However, subgroup analyses were made based on stent types or drugs, and consistent results were observed. Third, the included randomized trials might not be powered for certain end points or lacked multiple adjustments in statistical hierarchy. However, there was consistency between direct and indirect estimate analyses, suggesting that the overall effect is relatively robust. Fourth, although network meta-analysis respects randomization, it does not represent randomized evidence, as there are indirect comparisons of strategies that are not compared head to head.

## 6. Conclusions

In conclusion, among patients treated with DESs, 3-month DAPT followed by P2Y12 inhibitor monotherapy reduced the risks of net clinical events and bleeding events without increasing ischemic events, and the benefits were consistent for male patients with ACS, complex lesions, single-vessel disease, young age, and low BMI. Although DAPT beyond 1 year significantly reduced the risk of myocardial infarction, the risk of major bleeding was also increased. Taken together, 3-month DAPT followed by P2Y12 inhibitor monotherapy may be the optimal antiplatelet strategy for patients receiving DESs.

## Figures and Tables

**Table 1:** Comparisons for Primary Outcomes of All DAPT Strategies.

	**Net Clinical Events**					
Ischemic events	1-month DAPT (P2Y12 inhibitor)	1.13 (0.66, 1.99)	1.56 (0.93, 2.67)	1.62 (0.98, 2.72)	1.58 (0.99, 2.57)	1.58 (0.94, 2.65)
	1.01 (0.64, 1.68)	3-month DAPT (P2Y12 inhibitor)	1.38 (0.97, 1.96)	1.42 (1.03, 1.98)	1.39 (1.06, 1.82)	1.39 (0.99, 1.94)
	0.93 (0.58, 1.61)	0.92 (0.58, 1.48)	3-month DAPT (aspirin)	1.04 (0.77, 1.38)	1.01 (0.81, 1.27)	1.01 (0.75, 1.37)
	0.87 (0.56, 1.36)	0.86 (0.56, 1.27)	0.93 (0.59, 1.39)	6-month DAPT (aspirin)	0.98 (0.81, 1.18)	0.98 (0.79, 1.19)
	0.94 (0.67, 1.39)	0.93 (0.68, 1.29)	1.01 (0.71, 1.41)	1.09 (0.86, 1.43)	12-month DAPT	1.00 (0.81, 1.22)
	0.95 (0.60, 1.44)	0.94 (0.61, 1.36)	1.01 (0.64, 1.49)	1.09 (0.83, 1.39)	1 (0.76, 1.25)	>12-month DAPT
	**Myocardial Infarction**					
Major bleeding	1-month DAPT (P2Y12 inhibitor)	0.84 (0.43, 1.43)	1.09 (0.57, 1.97)	1.08 (0.65, 1.85)	0.98 (0.63, 1.49)	0.65 (0.40, 1.14)
	1.27 (0.42, 2.49)	3-month DAPT (P2Y12 inhibitor)	1.31 (0.73, 2.45)	1.30 (0.82, 2.31)	1.18 (0.81, 1.86)	0.78 (0.50, 1.43)
	1.27 (0.36, 3.60)	1.02 (0.37, 3.16)	3-month DAPT (aspirin)	1.00 (0.59, 1.77)	0.90 (0.58, 1.43)	0.60 (0.36, 1.09)
	0.86 (0.32, 1.68)	0.68 (0.35, 1.43)	0.67 (0.23, 1.80)	6-month DAPT (aspirin)	0.90 (0.67, 1.19)	0.61 (0.44, 0.86)
	0.73 (0.29, 1.19)	0.57 (0.34, 1.00)	0.56 (0.21, 1.36)	0.84 (0.53, 1.30)	12-month DAPT	0.67 (0.51, 0.93)
	0.42 (0.14, 0.78)	0.33 (0.17, 0.66)	0.33 (0.11, 0.87)	0.49 (0.29, 0.78)	0.58 (0.37, 0.89)	>12-month DAPT

**Table 2:** Comparisons for Secondary Outcomes of All DAPT Strategies.

	**All-Cause Death**					
Cardiac death	1-month DAPT (P2Y12 inhibitor)	0.90 (0.51, 1.52)	1.10 (0.61, 1.91)	0.97 (0.55, 1.49)	1.08 (0.71, 1.53)	1.15 (0.67, 1.72)
	1.15 (0.40, 3.44)	3-month DAPT (P2Y12 inhibitor)	1.22 (0.69, 2.20)	1.07 (0.63, 1.73)	1.19 (0.81, 1.78)	1.28 (0.77, 2.00)
	0.81 (0.27, 2.45)	0.70 (0.36, 1.37)	3-month DAPT (aspirin)	0.88 (0.50, 1.48)	0.98 (0.64, 1.49)	1.04 (0.61, 1.68)
	0.87 (0.30, 2.50)	0.76 (0.42, 1.37)	1.07 (0.58, 2.02)	6-month DAPT (aspirin)	1.12 (0.83, 1.56)	1.18 (0.90, 1.57)
	0.81 (0.30, 2.18)	0.71 (0.44, 1.11)	1.00 (0.62, 1.66)	0.94 (0.64, 1.35)	12-month DAPT	1.06 (0.79, 1.36)
	0.83 (0.29, 2.34)	0.72 (0.41, 1.27)	1.02 (0.57, 1.87)	0.95 (0.68, 1.34)	1.01 (0.73, 1.41)	>12-month DAPT
	**Stroke**					
Definite or probable stent thrombosis	1-month DAPT (P2Y12 inhibitor)	1.75 (0.82, 4.10)	1.00 (0.38, 2.75)	1.27 (0.69, 2.59)	1.18 (0.74, 2.17)	1.31 (0.74, 2.72)
	1.22 (0.36, 4.58)	3-month DAPT (P2Y12 inhibitor)	0.57 (0.20, 1.56)	0.73 (0.35, 1.49)	0.68 (0.37, 1.24)	0.75 (0.37, 1.53)
	0.97 (0.30, 3.86)	0.80 (0.25, 2.86)	3-month DAPT (aspirin)	1.27 (0.52, 3.20)	1.19 (0.52, 2.74)	1.32 (0.55, 3.27)
	1.07 (0.38, 3.36)	0.88 (0.31, 2.46)	1.10 (0.38, 2.97)	6-month DAPT (aspirin)	0.94 (0.63, 1.37)	1.03 (0.69, 1.56)
	1.23 (0.52, 3.54)	1.01 (0.44, 2.56)	1.27 (0.54, 2.97)	1.16 (0.69, 2.04)	12-month DAPT	1.10 (0.78, 1.61)
	2.08 (0.72, 6.55)	1.69 (0.60, 4.87)	2.13 (0.72, 5.87)	1.92 (1.03, 3.71)	1.67 (0.89, 2.92)	>12-month DAPT
	**TVR/TLR**					
Revascularization	1-month DAPT (P2Y12 inhibitor)	0.81 (0.24, 2.38)	1.17 (0.60, 1.93)	1.37 (0.75, 2.23)	1.02 (0.60, 1.42)	0.87 (0.36, 1.66)
	1.28 (0.45, 3.94)	3-month DAPT (P2Y12 inhibitor)	1.43 (0.46, 4.58)	1.69 (0.56, 5.42)	1.23 (0.43, 3.71)	1.07 (0.31, 3.58)
	0.86 (0.58, 1.42)	0.68 (0.23, 1.97)	3-month DAPT (aspirin)	1.17 (0.71, 2.04)	0.87 (0.59, 1.27)	0.75 (0.34, 1.47)
	0.87 (0.58, 1.33)	0.68 (0.22, 1.93)	1.01 (0.64, 1.52)	6-month DAPT (aspirin)	0.74 (0.51, 1.04)	0.64 (0.30, 1.20)
	1.01 (0.79, 1.46)	0.81 (0.28, 2.21)	1.18 (0.85, 1.63)	1.17 (0.90, 1.59)	12-month DAPT	0.86 (0.44, 1.54)
	0.92 (0.63, 1.55)	0.73 (0.24, 2.10)	1.07 (0.70, 1.70)	1.06 (0.74, 1.66)	0.90 (0.68, 1.25)	>12-month DAPT
	**Any bleeding**					
	1-month DAPT (P2Y12 inhibitor)	2.3 (0.80, 7.48)	2.96 (0.99, 9.85)	3.09 (1.07, 9.82)	3.99 (1.46, 12.23)	5.94 (2.00, 18.66)
		3-month DAPT (P2Y12 inhibitor)	1.29 (0.71, 2.28)	1.35 (0.79, 2.14)	1.74 (1.20, 2.49)	2.59 (1.47, 4.05)
			3-month DAPT (aspirin)	1.05 (0.59, 1.82)	1.35 (0.86, 2.17)	2.01 (1.10, 3.47)
				6-month DAPT (aspirin)	1.29 (0.95, 1.87)	1.91 (1.32, 2.71)
					12-month DAPT	1.48 (1.00, 2.04)
						>12-month DAPT

**Table 3:** Subgroup Analyses of Primary Outcomes with Six DAPT Strategies.

	**ACS**					
Stable angina	1-month DAPT (P2Y12 inhibitor)	0.72 (0.45, 1.30)	1.27 (0.77, 2.35)	1.09 (0.66, 1.85)	1.21 (0.83, 1.86)	0.85 (0.44, 1.65)
	0.90 (0.45, 1.51)	3-month DAPT (P2Y12 inhibitor)	1.76 (1.05, 2.87)	1.50 (0.92, 2.27)	1.67 (1.15, 2.25)	1.18 (0.60, 2.09)
	0.87 (0.48, 1.50)	0.97 (0.55, 1.89)	3-month DAPT (aspirin)	0.85 (0.51, 1.36)	0.95 (0.63, 1.35)	0.67 (0.33, 1.23)
	0.70 (0.39, 1.13)	0.78 (0.45, 1.42)	0.80 (0.46, 1.38)	6-month DAPT (aspirin)	1.11 (0.82, 1.52)	0.79 (0.49, 1.21)
	0.80 (0.52, 1.12)	0.89 (0.59, 1.45)	0.91 (0.59, 1.37)	1.14 (0.80, 1.63)	12-month DAPT	0.71 (0.41, 1.17)
	0.85 (0.41, 1.63)	0.95 (0.47, 2.05)	0.98 (0.48, 1.96)	1.22 (0.68, 2.16)	1.07 (0.60, 1.90)	>12-month DAPT
	**Young Patients**					
Old patients	1-month DAPT (P2Y12 inhibitor)	0.79 (0.49, 1.44)	1.05 (0.63, 1.86)	1.41 (0.91, 2.49)	1.12 (0.79, 1.65)	0.84 (0.54, 1.59)
	1.01 (0.43, 2.02)	3-month DAPT (P2Y12 inhibitor)	1.33 (0.75, 2.22)	1.78 (1.09, 2.93)	1.42 (0.94, 2.01)	1.07 (0.65, 1.84)
	0.62 (0.24, 1.44)	0.62 (0.28, 1.42)	3-month DAPT (aspirin)	1.34 (0.83, 2.30)	1.06 (0.72, 1.57)	0.80 (0.50, 1.45)
	0.80 (0.38, 1.60)	0.79 (0.44, 1.55)	1.30 (0.61, 2.90)	6-month DAPT (aspirin)	0.79 (0.55, 1.08)	0.60 (0.41, 0.90)
	0.64 (0.33, 1.10)	0.63 (0.40, 1.03)	1.03 (0.53, 2.01)	0.79 (0.52, 1.17)	12-month DAPT	0.75 (0.55, 1.20)
	0.87 (0.39, 1.94)	0.87 (0.46, 1.91)	1.42 (0.64, 3.47)	1.09 (0.67, 1.92)	1.38 (0.84, 2.46)	>12-month DAPT
	**Diabetes**					
No diabetes	1-month DAPT (P2Y12 inhibitor)	1.00 (0.57, 1.87)	1.17 (0.62, 2.21)	1.54 (0.88, 2.75)	1.33 (0.86, 2.11)	1.32 (0.74, 2.40)
	1.37 (0.70, 2.10)	3-month DAPT (P2Y12 inhibitor)	1.16 (0.63, 2.06)	1.54 (0.89, 2.57)	1.32 (0.88, 1.92)	1.31 (0.74, 2.23)
	0.78 (0.44, 1.24)	0.57 (0.37, 0.98)	3-month DAPT (aspirin)	1.33 (0.75, 2.38)	1.14 (0.73, 1.80)	1.13 (0.62, 2.08)
	0.92 (0.53, 1.42)	0.68 (0.44, 1.11)	1.18 (0.74, 1.87)	6-month DAPT (aspirin)	0.86 (0.60, 1.23)	0.85 (0.56, 1.31)
	0.83 (0.52, 1.16)	0.61 (0.45, 0.92)	1.07 (0.76, 1.52)	0.91 (0.68, 1.23)	12-month DAPT	0.99 (0.66, 1.46)
	1.30 (0.70, 2.04)	0.95 (0.60, 1.58)	1.67 (1.00, 2.65)	1.41 (0.97, 2.01)	1.56 (1.07, 2.13)	>12-month DAPT
	**Prasugrel/Ticagrelor**					
Clopidogrel	1-month DAPT (P2Y12 inhibitor)	0.88 (0.17, 7.07)	1.36 (0.14, 12.95)	1.02 (0.06, 17.86)	1.16 (0.23, 5.68)	0.61 (0.06, 6.02)
	0.85 (0.46, 1.60)	3-month DAPT (P2Y12 inhibitor)	1.52 (0.18, 8.20)	1.10 (0.07, 12.21)	1.29 (0.39, 2.90)	0.69 (0.09, 3.80)
	0.93 (0.62, 1.42)	1.09 (0.60, 1.98)	3-month DAPT (aspirin)	0.75 (0.05, 13.66)	0.85 (0.17, 4.43)	0.45 (0.05, 4.64)
	0.81 (0.56, 1.18)	0.95 (0.53, 1.66)	0.88 (0.63, 1.20)	6-month DAPT (aspirin)	1.15 (0.11, 12.04)	0.60 (0.11, 3.52)
	0.87 (0.63, 1.20)	1.02 (0.59, 1.71)	0.93 (0.72, 1.21)	1.07 (0.89, 1.29)	12-month DAPT	0.52 (0.10, 2.73)
	0.92 (0.62, 1.33)	1.08 (0.60, 1.86)	0.99 (0.70, 1.35)	1.13 (0.88, 1.45)	1.06 (0.86, 1.28)	>12-month DAPT
	**New-Generation DESs**					
First-generation DESs	1-month DAPT (P2Y12 inhibitor)	1.01 (0.67, 1.66)	1.22 (0.84, 1.94)	1.26 (0.92, 1.87)	1.23 (0.97, 1.72)	1.12 (0.78, 1.70)
	-	3-month DAPT (P2Y12 inhibitor)	1.22 (0.76, 1.90)	1.25 (0.83, 1.87)	1.22 (0.86, 1.70)	1.11 (0.70, 1.69)
	-	-	3-month DAPT (aspirin)	1.03 (0.71, 1.51)	1.01 (0.75, 1.37)	0.91 (0.60, 1.38)
	-	-	-	6-month DAPT (aspirin)	0.98 (0.79, 1.21)	0.88 (0.68, 1.14)
	-	-	-	2.48 (0.79, 8.43)	12-month DAPT	0.90 (0.69, 1.19)
	-	-	-	4.63 (1.05, 21.99)	1.87 (0.71, 4.97)	>12-month DAPT
	**Caucasians**					
No Caucasian	1-month DAPT (P2Y12 inhibitor)	0.72 (0.45, 1.32)	1.27 (0.77, 2.36)	1.09 (0.66, 1.83)	1.21 (0.82, 1.86)	0.85 (0.44, 1.62)
	0.88 (0.47, 1.66)	3-month DAPT (P2Y12 inhibitor)	1.76 (1.05, 2.89)	1.5 (0.92, 2.3)	1.68 (1.15, 2.25)	1.18 (0.59, 2.09)
	0.65 (0.3, 1.41)	0.74 (0.38, 1.42)	3-month DAPT (aspirin)	0.85 (0.5, 1.36)	0.95 (0.63, 1.34)	0.67 (0.33, 1.22)
	0.55 (0.29, 1.02)	0.62 (0.39, 0.97)	0.84 (0.44, 1.59)	6-month DAPT (aspirin)	1.12 (0.82, 1.52)	0.79 (0.49, 1.2)
	0.63 (0.37, 1.08)	0.72 (0.52, 1.01)	0.98 (0.55, 1.69)	1.16 (0.86, 1.58)	12-month DAPT	0.7 (0.41, 1.16)
	0.56 (0.3, 1.07)	0.64 (0.41, 1.06)	0.87 (0.46, 1.68)	1.04 (0.78, 1.43)	0.89 (0.65, 1.28)	>12-month DAPT

## Data Availability

The data presented in this study are available on request from the corresponding author.

## Supplementary Materials

The following supporting information can be downloaded at: https://www.mdpi.com/article/10.3390/jcm11154465/s1, Figure S1: PRISMA Diagram for Study Inclusion; Figure S2: Methodological Quality Assessment for Each Included Randomized Trial; Figure S3: Network Evidence Plot for Secondary Outcomes; Figure S4: Funnel Plot of Publication Bias for Primary and Secondary Outcomes; Figure S5: Network Node-Split for Primary and Secondary Outcomes; Figure S6: Subgroup Analyses of Primary Outcomes with Five DAPT Strategies; Figure S7: Subgroup Analyses of Primary Outcomes with Four DAPT Strategies; Table S1: Baseline Characteristics of Included Randomized Trials; Table S2: Subgroup Analyses of Primary Outcomes with Five DAPT Strategies; Table S3: Subgroup Analyses of Primary Outcomes with Four DAPT Strategies.
