# Supplementary material for: The Optimal Strategy of Dual Antiplatelet Therapy after Percutaneous Coronary Intervention with Drug-Eluting Stent
Source: J Clin Med. 2022 Jul 31;11(15):4465. doi: 10.3390/jcm11154465 (PMC9370028; doi:10.3390/jcm11154465)
Supplement: Supplementary file 1 [file jcm-11-04465-s001.zip › jcm-1815816-supplementary.pdf]

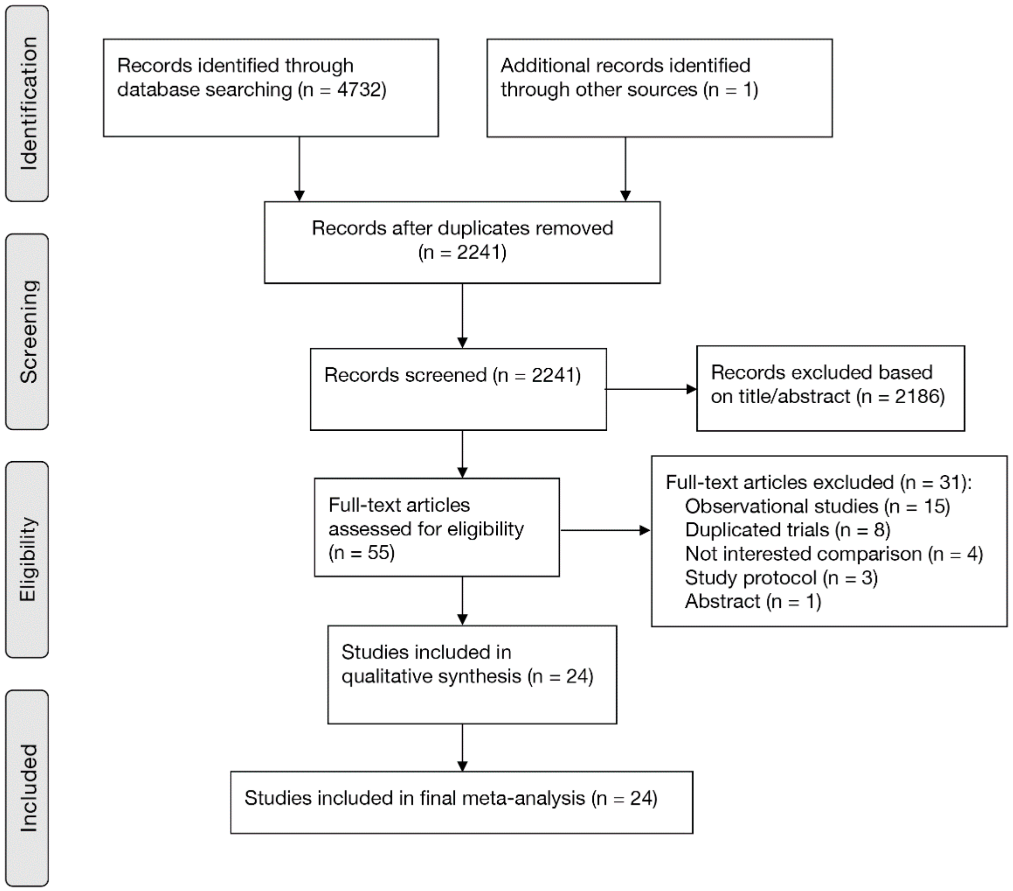

Supplementary Figure S1. PRISMA Diagram for Study Inclusion

	Random sequence generation (selection bias)	Allocation concealment (selection bias)	Blinding of participants and personnel (performance bias)	Blinding of outcome assessment (detection bias)	Incomplete outcome data (attrition bias)	Selective reporting (reporting bias)	Other bias
ARCTIC-Interruption	+	+	+	?	+	+	?
DAPT-STEMI	?	?	−	+	+	+	+
DAPT Study	?	?	+	−	+	+	−
DES LATE	+	?	−	−	+	+	?
EXCELLENT	+	+	−	−	+	+	?
GLOBAL LEADERS	+	+	−	+	+	+	+
I-LOVE-IT 2	+	−	?	+	+	+	−
ISAR-SAFE	+	+	+	?	+	+	−
ITALIC	+	?	−	?	+	+	−
IVUS-XPL	+	+	−	+	+	?	−
NIPPON	+	+	−	?	+	+	−
OPTIDUAL	+	+	−	+	+	+	−
OPTIMA-C	+	+	−	+	+	+	?
OPTIMIZE	+	+	−	+	+	+	−
PRODIGY	+	+	?	+	?	+	?
REAL-ZEST LATE	+	+	−	−	+	+	?
REDUCE	+	?	−	+	+	+	+
RESET	+	+	−	?	+	+	?
SECURITY	+	+	?	?	?	+	−
SMART-CHOICE	+	?	−	+	+	+	+
SMART-DATE	+	+	−	+	+	+	?
STOPDAPT-2	+	?	−	+	+	+	+
TICO	+	+	−	?	+	+	+
TWILIGHT	+	+	−	?	+	+	+

Supplementary Figure S2. Methodological Quality Assessment for Each Included Randomized Trial. +, low risk of bias; -, high risk of bias; ?, unclear risk of bias

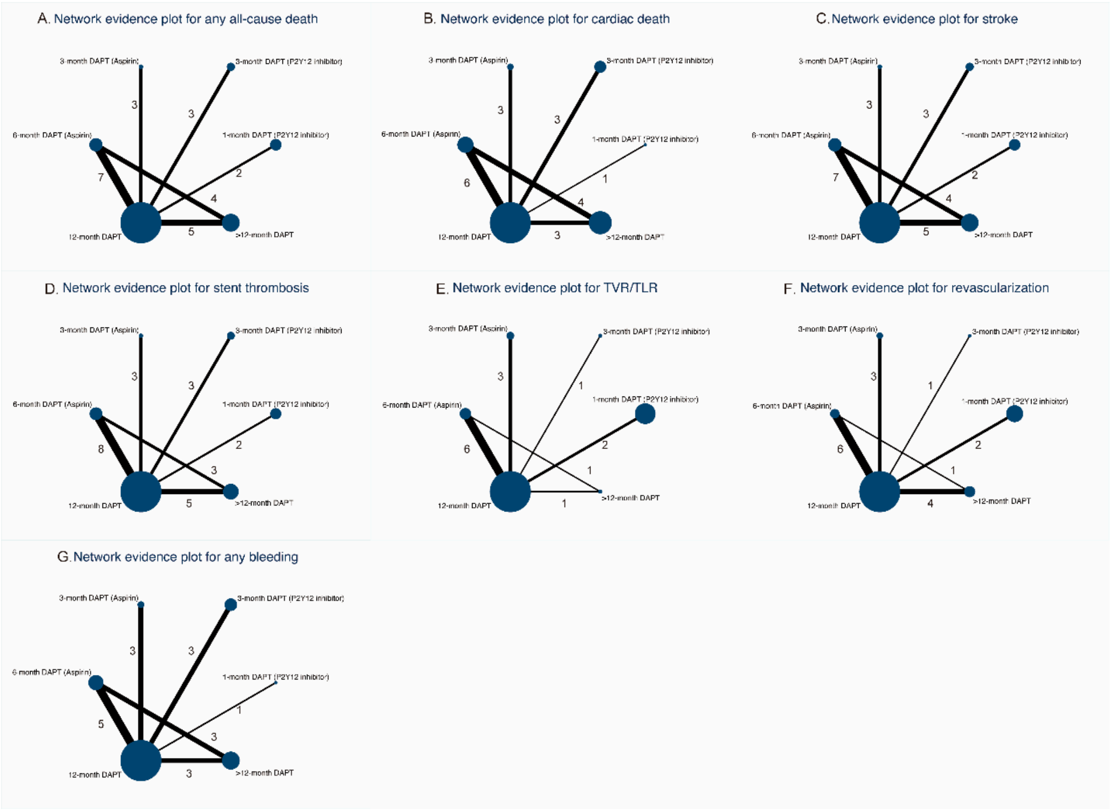

Supplementary Figure S3. Network Evidence Plot for Secondary Outcomes

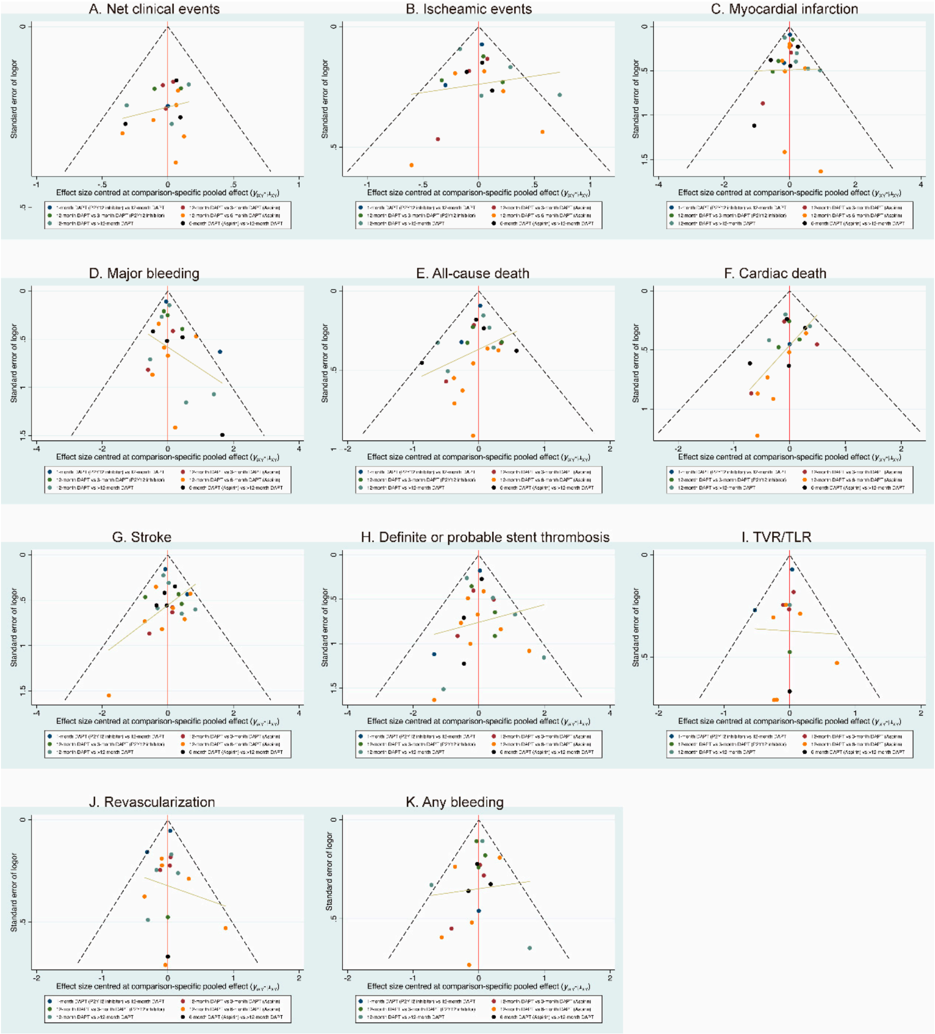

Supplementary Figure S4. Funnel Plot of Publication Bias for Primary and Secondary Outcomes

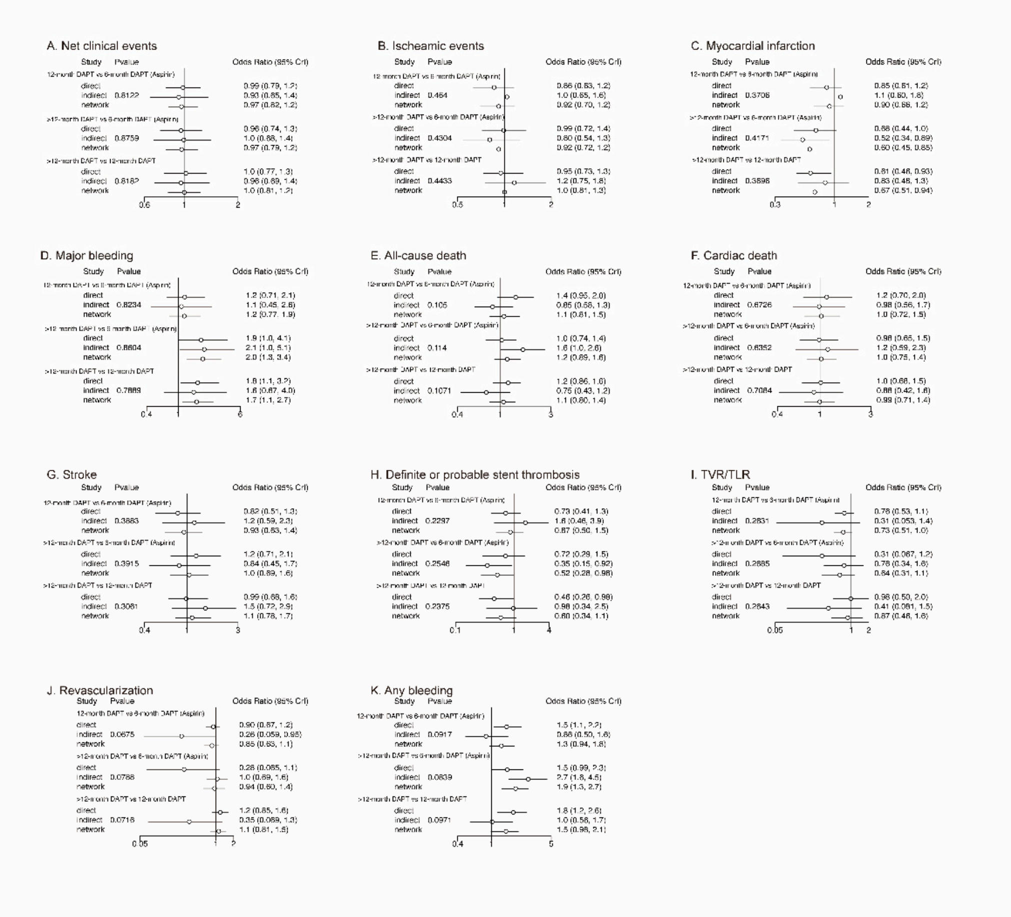

Supplementary Figure S5. Network Node-Split for Primary and Secondary Outcomes

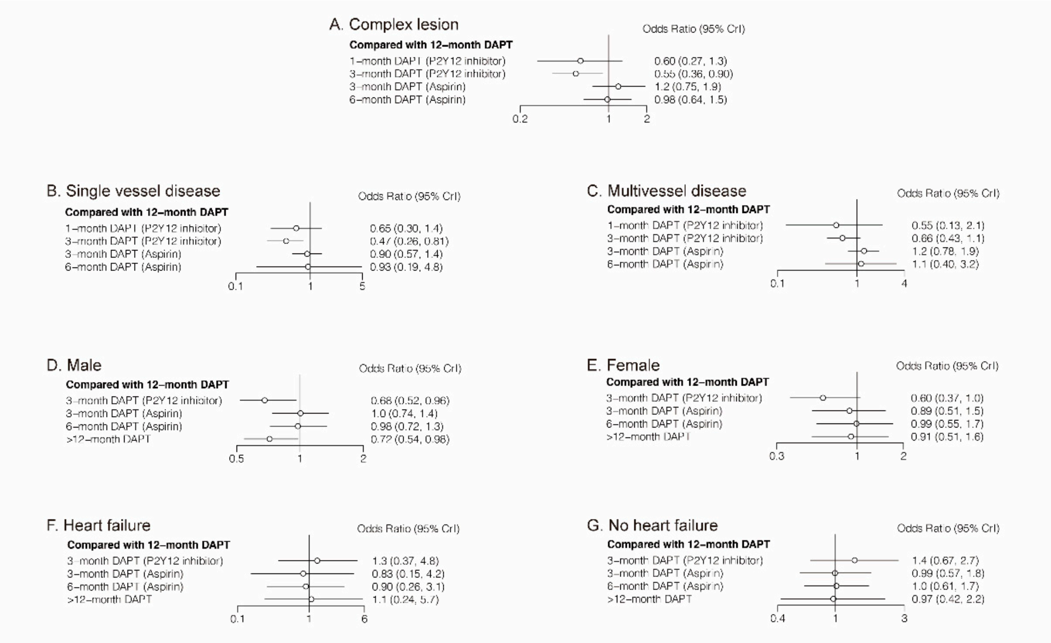

Supplementary Figure S6. Subgroup Analyses of Primary Outcomes with Five DAPT Strategies

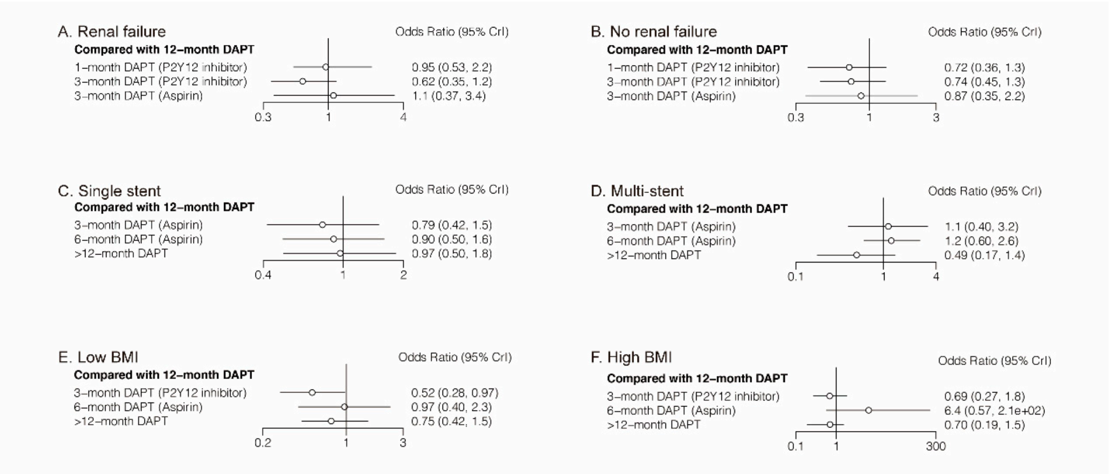

Supplementary Figure S7. Subgroup Analyses of Primary Outcomes with Four DAPT Strategies

Supplementary Table S1. Baseline Characteristics of Included Randomized Trials

	Nation	Study Period	Number of patients	DAPT regimen	Continued drug	Mean age, years	Male (%)	ACS (%)	Diabetes (%)	Stent type	Follow-up time
1 month vs 12 months											
GLOBAL LEADERS[1]	worldwide	2013.07-2015.11	7980/7988	Aspirin (75-100mg/d) +clopidogrel (75mg/d)/ticagrelor (90mg twice per day)	ticagrelor	NA	76.6/76.9	47.0/46.8	25.7/24.9	BES	24 months
STOPDAPT-2[2]	Japan	2015.12-2017.12	1500/1509	Aspirin (81-200mg/d) +clopidogrel (75mg/d) /prasugrel (3.75mg/d)	clopidogrel	68.1/69.1	78.9/76.5	37.7/38.6	39.0/38.0	EES	12 months

3 months vs 12 months											
OPTIMIZE[3]	Brazil	2010.04-2012.03	1563/1556	Aspirin (100-200mg/d) +clopidogrel (75mg/d)	Aspirin	61.3/61.9	64.5/63.1	31.6/32.3	35.4/35.3	ZES	12 months
RESET[4]	South Korea	2009.04-2010.12	1059/1058	Aspirin (100mg/d) +clopidogrel (75mg/d)	Aspirin	62.4/62.4	64.4/62.9	55.6/53.6	29.9/28.8	E-ZES/SES/EES	12 months
TWILIGHT[5]	worldwide	2015.07-2017.12	3555/3564	Aspirin (81-100mg/d) +ticagrelor (90mg/d)	ticagrelor	65.2/65.1	76.2/76.1	63.9/65.7	37.1/36.5	NA	12 months
REDUCE[6]	worldwide	2014.06=2016.05	751/745	Aspirin +clopidogrel (75mg/d) /prasugrel (10 mg/d) / ticagrelor (180mg/d)	Aspirin	61.0/60.0	82.6/77.3	100/100	21.6/19.5	SES	12 months
SMART-CHOICE[7]	South Korea	2014.03-2017.07	1495/1498	Aspirin(100mg/d) +clopidogrel (75mg/d) /prasugrel (10 mg/d) / ticagrelor (90mg twice per day)	P2Y12 Inhibitor	64.6/64.4	72.7/74.2	58.2/58.2	38.2/36.8	EES/SES	12 months
TICO[8]	South Korea	2015.08-2018.10	1527/1529	Aspirin(100mg/d) +ticagrelor (90mg twice per day)	ticagrelor	61/61	79/80	100/100	27/27	SES	12 months
6 months vs 12 months											
OPTIMA-C[9]	South Korea	2011.04-2014.05	683/684	Aspirin (100mg/d) +clopidogrel (75mg/d)	Aspirin	62.8/64.4	70.0/67.8	50.4/50.9	29.1/29.7	BES/ZES	12 months
I-LOVE-IT 2[10]	China	NA	909/920	Aspirin (100mg/d) +clopidogrel (75mg/d)	Aspirin	60.4/60	67.2/68.7	82.7/80.9	23.2/22.1	BP-SES	18 months
IVUS-XPL[11]	South Korea	2010.10-2014.07	699/701	Aspirin (100mg/d) +clopidogrel (75mg/d)	Aspirin	63/64	67.2/70.5	49.1/48.9	35.6/36.7	EES	12 months
ISAR-SAFE[12]	worldwide	2008.10-2014.04	1997/2003	Aspirin (81-200mg/d) +clopidogrel (75mg/d)	Aspirin	67.2/67.2	83.2/80.5	39.8/40.3	24.8/24.2	PES/SES/EES/ZES/BES/BMS	9 months
SECURITY[13]	worldwide	2009.07-2014.06	682/717	Aspirin + clopidogrel (75mg/d)	Aspirin	64.9/65.5	77.6/76.8	31.2/31.9	30.2/31.1	ZES/BES/EES	24 months

EXCELLENT[14]	South Korea	2008.06-2009.07	722/721	Aspirin (75-100mg/d) +clopidogrel (75mg/d)	Aspirin	63/62.4	65.1/63.9	51.1/52.0	37.7/38.6	EES/SES	12 months
DAPT-STEMI[15]	Netherlands, Norway, Poland, and Switzerland	2011.12-2015.06	433/437	Aspirin (100mg/d) +clopidogrel (75mg/d) /prasugrel (10 mg/d) / ticagrelor (90mg twice per day)	Aspirin	59.8/60.2	78/76	100/100	13/14	ZES	24 months
6 months vs 12.6-18 months											
SMART-DATE[16]	South Korea	2012.09-2015.12	1357/1355	Aspirin (100mg/d) +clopidogrel (75mg/d) /prasugrel (10 mg/d) / ticagrelor (90mg twice per day)	Aspirin	62.0/62.2	74.9/75.9	100/100	26.9/28.1	EES/ZES/BES	18 months
6 months vs 18 months											
NIPPON[17]	Japan	2011.12-2015.06	1654/1653	Aspirin (81-162mg/d) +clopidogrel (75mg/d) /ticlopidine (200mg/d)	Aspirin	67.4/67.2	78.8/79.4	37.4/38.4	31.9/33.4	BES	18 months
6 months vs 24 months											
ITALIC[18]	European and Middle East	2008.11-2013.12	926/924	Aspirin + clopidogrel (75mg/d)/prasugrel (10mg/d) /ticagrelor (90mg twice per day)	Aspirin	61.6/61.5	81.0/79.3	43.2/43.9	36.3/37.8	EES	24 months
PRODIGY[19]	Italy	2006.12-2008.12	983/987	Aspirin (80-160mg/d) +clopidogrel (75mg/d)	Aspirin	67.9 67.8	76.0/77.4	74.6/71.1	23.7/24.7	EES/PES/ZES/ BMS	24 months

12 months vs 18-30 months											
ARCTIC-Interruption[20]	France	2011.01-2012.03	624/635	Aspirin + clopidogrel (75-150mg/d) /prasugrel (10 mg/d)	-	64/64	80.6/80.0	26.8/24.6	35.6/31.1	First /second generation DES	17 months
12 months vs 30 months											
DAPT Study[21]	worldwide	2009.08-2011.07	4941/5050	Aspirin (75-162mg/d) +clopidogrel (75mg/d) /prasugrel (10 or 5mg/d)	-	61.6/61.8	74.0/75.3	42.6/42.8	30.0/31.0	EES/PES/ZES/ SES	33 months
12 months vs 36 months											
DES LATE[22]	South Korea	2007.07-2011.07	2514/2531	Aspirin(100-200mg/d) +clopidogrel (75mg/d)	-	62.3/62.5	69.6/69.1	61.7/59.7	28.2/28.0	SES/PES/ZES/ EES/others	24 months
REAL-ZEST LATE[23]	South Korea	2007.07-2008.09	1344/1357	Aspirin (100-200mg/d) +clopidogrel (75mg/d)	-	61.9/62.0	69.4/70.0	62.8/62.1	27.1/25.1	SES/PES/ZES/ other	24 months
12 months vs 18-48 months											
OPTIDUAL[24]	France	2009.01-2013.01	690/695	Aspirin (75-160mg/d) +clopidogrel (75mg/d)	-	64.2/64.1	79.3/81.7	38.0/34.4	32.2/30.6	SES/PES/ZES/ EES	36 months

ACS: acute coronary syndrome; BES: Biolimus-eluting stent; BMS: baremetal stent; DAPT: dual antiplatelet therapy; EES: Everolimus-eluting stents; PES: Paclitaxel-eluting stent; SES: Sirolimus-eluting stent; ZES: Zotarolimus-eluting stent

Supplementary Table S2. Subgroup Analyses of Primary Outcomes with Five DAPT Strategies

	Complex lesion				
	1-month DAPT (P2Y12 inhibitor)	0.92 (0.39, 2.35)	2 (0.81, 5.07)	1.64 (0.69, 4)	1.67 (0.78, 3.66)
	-	3-month DAPT (P2Y12 inhibitor)	2.17 (1.09, 4.08)	1.78 (0.94, 3.23)	1.82 (1.11, 2.78)
	-	-	3-month DAPT (Aspirin)	0.82 (0.43, 1.56)	0.84 (0.52, 1.34)
	-	-	-	6-month DAPT (Aspirin)	1.02 (0.66, 1.57)
	-	-	-	-	12-month DAPT
	Single vessel disease				
Multivessel disease	1-month DAPT (P2Y12 inhibitor)	0.73 (0.27, 1.87)	1.4 (0.57, 3.5)	1.44 (0.24, 8.9)	1.55 (0.71, 3.38)
	0.83 (0.18, 3.38)	3-month DAPT (P2Y12 inhibitor)	1.92 (0.95, 3.96)	1.97 (0.37, 11.41)	2.11 (1.24, 3.77)
	0.45 (0.1, 1.84)	0.53 (0.29, 1.07)	3-month DAPT (Aspirin)	1.03 (0.2, 5.67)	1.11 (0.7, 1.74)
	0.48 (0.08, 2.63)	0.59 (0.19, 1.84)	1.09 (0.35, 3.33)	6-month DAPT (Aspirin)	1.08 (0.21, 5.25)
	0.55 (0.13, 2.1)	0.66 (0.43, 1.1)	1.23 (0.78, 1.91)	1.13 (0.4, 3.15)	12-month DAPT
	Male				
Female	3-month DAPT (P2Y12 inhibitor)	1.48 (0.93, 2.22)	1.44 (0.9, 2.15)	1.47 (1.04, 1.92)	1.05 (0.68, 1.55)
	0.68 (0.33, 1.48)	3-month DAPT (Aspirin)	0.97 (0.64, 1.5)	0.99 (0.73, 1.35)	0.71 (0.47, 1.1)

	0.6 (0.29, 1.38)	0.89 (0.41, 1.97)	6-month DAPT (Aspirin)	1.02 (0.75, 1.39)	0.73 (0.53, 1.02)
	0.6 (0.36, 1.02)	0.89 (0.5, 1.51)	0.99 (0.54, 1.73)	12-month DAPT	0.72 (0.54, 0.98)
	0.65 (0.32, 1.44)	0.97 (0.43, 2.11)	1.08 (0.58, 1.97)	1.09 (0.63, 1.92)	>12-month DAPT
	Heart failure				
No heart failure	3-month DAPT (P2Y12 inhibitor)	0.64 (0.07, 5.05)	0.69 (0.11, 4.03)	0.77 (0.21, 2.67)	0.82 (0.11, 6.66)
	1.37 (0.56, 3.36)	3-month DAPT (Aspirin)	1.09 (0.14, 8.88)	1.21 (0.24, 6.52)	1.32 (0.14, 14.05)
	1.33 (0.56, 3.12)	0.98 (0.46, 2.07)	6-month DAPT (Aspirin)	1.11 (0.32, 3.88)	1.19 (0.49, 3.37)
	1.36 (0.67, 2.73)	0.99 (0.57, 1.76)	1.02 (0.61, 1.7)	12-month DAPT	1.08 (0.24, 5.68)
	1.4 (0.48, 4.14)	1.03 (0.38, 2.79)	1.05 (0.55, 2.03)	1.03 (0.46, 2.37)	>12-month DAPT

Supplementary Table S3. Subgroup Analyses of Primary Outcomes with Four DAPT Strategies

	Renal failure			
No renal failure	1-month DAPT (P2Y12 inhibitor)	0.65 (0.24, 1.52)	1.12 (0.27, 3.97)	1.04 (0.45, 1.9)
	0.97 (0.39, 2.11)	3-month DAPT (P2Y12 inhibitor)	1.75 (0.48, 6.18)	1.6 (0.85, 2.82)
	0.82 (0.25, 2.43)	0.84 (0.3, 2.53)	3-month DAPT (Aspirin)	0.91 (0.3, 2.78)
	0.72 (0.36, 1.32)	0.74 (0.45, 1.31)	0.87 (0.35, 2.21)	12-month DAPT
	Single stent			
Multi-stent	3-month DAPT (Aspirin)	1.13 (0.48, 2.69)	1.27 (0.66, 2.39)	1.22 (0.49, 3)
	0.92 (0.25, 3.22)	6-month DAPT (Aspirin)	1.11 (0.62, 1.99)	1.07 (0.57, 2.02)
	1.14 (0.4, 3.19)	1.23 (0.6, 2.61)	12-month DAPT	0.97 (0.5, 1.84)
	2.31 (0.53, 9.83)	2.49 (0.93, 7.08)	2.03 (0.74, 5.72)	>12-month DAPT
	Low BMI			
High BMI	3-month DAPT (P2Y12 inhibitor)	1.86 (0.61, 5.41)	1.93 (1.03, 3.57)	1.44 (0.64, 3.76)
	0.11 (0, 1.44)	6-month DAPT (Aspirin)	1.03 (0.43, 2.51)	0.78 (0.28, 2.45)
	0.69 (0.27, 1.83)	6.39 (0.57, 213.82)	12-month DAPT	0.75 (0.42, 1.52)
	0.98 (0.31, 5.36)	9.84 (0.74, 398.08)	1.43 (0.65, 5.28)	>12-month DAPT

1. Vranckx P, Valgimigli M, Jüni P, Hamm C, Steg PG, Heg D, van Es GA, McFadden EP, Onuma Y, van Meijeren C, Chichareon P, Benit E, Möllmann H, Janssens L, Ferrario M, Moschovitis A, Zurakowski A, Dominici M, Van Geuns RJ, Huber K, Slagboom T, Serruys PW and Windecker S. Ticagrelor plus aspirin for 1 month, followed by ticagrelor monotherapy for 23 months vs aspirin plus clopidogrel or ticagrelor for 12 months, followed by aspirin monotherapy for 12 months after implantation of a drug-eluting stent: a multicentre, open-label, randomised superiority trial. Lancet. 2018;392:940-949.

2. Watanabe H, Domei T, Morimoto T, Natsuaki M, Shiomi H, Toyota T, Ohya M, Suwa S, Takagi K, Nanasato M, Hata Y, Yagi M, Suematsu N, Yokomatsu T, Takamisawa I, Doi M, Noda T, Okayama H, Seino Y, Tada T, Sakamoto H, Hibi K, Abe M, Kawai K, Nakao K, Ando K, Tanabe K, Ikari Y, Hanaoka KI, Morino Y, Kozuma K, Kadota K, Furukawa Y, Nakagawa Y and Kimura T. Effect of 1-Month Dual Antiplatelet Therapy Followed by Clopidogrel vs 12-Month Dual Antiplatelet Therapy on Cardiovascular and Bleeding Events in Patients Receiving PCI: The STOPDAPT-2 Randomized Clinical Trial. Jama. 2019;321:2414-2427.

3. Feres F, Costa RA, Abizaid A, Leon MB, Marin-Neto JA, Botelho RV, King SB, 3rd, Negoita M, Liu M, de Paula JE, Mangione JA, Meireles GX, Castello HJ, Jr., Nicolela EL, Jr., Perin MA, Devito FS, Labrunie A, Salvadori D, Jr., Gusmão M, Staico R, Costa JR, Jr., de Castro JP, Abizaid AS and Bhatt DL. Three vs twelve months of dual antiplatelet therapy after zotarolimus-eluting stents: the OPTIMIZE randomized trial. Jama. 2013;310:2510-22.

4. Kim BK, Hong MK, Shin DH, Nam CM, Kim JS, Ko YG, Choi D, Kang TS, Park BE, Kang WC, Lee SH, Yoon JH, Hong BK, Kwon HM and Jang Y. A new

strategy for discontinuation of dual antiplatelet therapy: the RESET Trial (REal Safety and Efficacy of 3-month dual antiplatelet Therapy following Endeavor zotarolimus-eluting stent implantation). J Am Coll Cardiol. 2012;60:1340-8.

5. Mehran R, Baber U, Sharma SK, Cohen DJ, Angiolillo DJ, Briguori C, Cha JY, Collier T, Dangas G, Dudek D, Džavík V, Escaned J, Gil R, Gurbel P, Hamm CW, Henry T, Huber K, Kastrati A, Kaul U, Kornowski R, Krucoff M, Kunadian V, Marx SO, Mehta SR, Moliterno D, Ohman EM, Oldroyd K, Sardella G, Sartori S, Shlofmitz R, Steg PG, Weisz G, Witzenbichler B, Han YL, Pocock S and Gibson CM. Ticagrelor with or without Aspirin in High-Risk Patients after PCI. N Engl J Med. 2019;381:2032-2042.

6. De Luca G, Damen SA, Camaro C, Benit E, Verdoia M, Rasoul S, Liew HB, Polad J, Ahmad WA, Zambahari R, Postma S, Kedhi E and Suryapranata H. Final results of the randomised evaluation of short-term dual antiplatelet therapy in patients with acute coronary syndrome treated with a new-generation stent (REDUCE trial). EuroIntervention. 2019;15:e990-e998.

7. Hahn JY, Song YB, Oh JH, Chun WJ, Park YH, Jang WJ, Im ES, Jeong JO, Cho BR, Oh SK, Yun KH, Cho DK, Lee JY, Koh YY, Bae JW, Choi JW, Lee WS, Yoon HJ, Lee SU, Cho JH, Choi WG, Rha SW, Lee JM, Park TK, Yang JH, Choi JH, Choi SH, Lee SH and Gwon HC. Effect of P2Y12 Inhibitor Monotherapy vs Dual Antiplatelet Therapy on Cardiovascular Events in Patients Undergoing Percutaneous Coronary Intervention: The SMART-CHOICE Randomized Clinical

Trial. Jama. 2019;321:2428-2437.

8. Kim BK, Hong SJ, Cho YH, Yun KH, Kim YH, Suh Y, Cho JY, Her AY, Cho S, Jeon DW, Yoo SY, Cho DK, Hong BK, Kwon H, Ahn CM, Shin DH, Nam CM, Kim JS, Ko YG, Choi D, Hong MK and Jang Y. Effect of Ticagrelor Monotherapy vs Ticagrelor With Aspirin on Major Bleeding and Cardiovascular Events in Patients With Acute Coronary Syndrome: The TICO Randomized Clinical Trial. Jama. 2020;323:2407-2416.

9. Lee BK, Kim JS, Lee OH, Min PK, Yoon YW, Hong BK, Shin DH, Kang TS, Kim BO, Cho DK, Jeon DW, Woo SI, Choi S, Kim YH, Kang WC, Kim S, Kim BK, Hong MK, Jang Y and Kwon HM. Safety of six-month dual antiplatelet therapy after second-generation drug-eluting stent implantation: OPTIMA-C Randomised Clinical Trial and OCT Substudy. EuroIntervention. 2018;13:1923-1930.

10. Han Y, Xu B, Xu K, Guan C, Jing Q, Zheng Q, Li X, Zhao X, Wang H, Zhao X, Li X, Yu P, Zang H, Wang Z, Cao X, Zhang J, Pang W, Li J, Yang Y and Dangas GD. Six Versus 12 Months of Dual Antiplatelet Therapy After Implantation of Biodegradable Polymer Sirolimus-Eluting Stent: Randomized Substudy of the I-LOVE-IT 2 Trial. Circ Cardiovasc Interv. 2016;9:e003145.

11. Hong SJ, Shin DH, Kim JS, Kim BK, Ko YG, Choi D, Her AY, Kim YH, Jang Y and Hong MK. 6-Month Versus 12-Month Dual-Antiplatelet Therapy Following Long Everolimus-Eluting Stent Implantation: The IVUS-XPL Randomized Clinical Trial. JACC Cardiovasc Interv. 2016;9:1438-46.

12. Schulz-Schüpke S, Byrne RA, Ten Berg JM, Neumann FJ, Han Y, Adriaenssens T, Tölg R, Seyfarth M, Maeng M, Zrenner B, Jacobshagen C, Mudra H, von Hodenberg E, Wöhrle J, Angiolillo DJ, von Merzljak B, Rifatov N, Kufner S, Morath T, Feuchtenberger A, Ibrahim T, Janssen PW, Valina C, Li Y, Desmet W, Abdel-Wahab M, Tiroch K, Hengstenberg C, Bernlochner I, Fischer M, Schunkert H, Laugwitz KL, Schömig A, Mehilli J and Kastrati A. ISAR-SAFE: a randomized, double-blind, placebo-controlled trial of 6 vs. 12 months of clopidogrel therapy after drug-eluting stenting. Eur Heart J. 2015;36:1252-63.

13. Colombo A, Chieffo A, Frasheri A, Garbo R, Masotti-Centol M, Salvatella N, Oteo Dominguez JF, Steffanon L, Tarantini G, Presbitero P, Menozzi A, Pucci E, Mauri J, Cesana BM, Giustino G and Sardella G. Second-generation drug-eluting stent implantation followed by 6- versus 12-month dual antiplatelet therapy: the SECURITY randomized clinical trial. J Am Coll Cardiol. 2014;64:2086-97.

14. Gwon HC, Hahn JY, Park KW, Song YB, Chae IH, Lim DS, Han KR, Choi JH, Choi SH, Kang HJ, Koo BK, Ahn T, Yoon JH, Jeong MH, Hong TJ, Chung WY, Choi YJ, Hur SH, Kwon HM, Jeon DW, Kim BO, Park SH, Lee NH, Jeon HK, Jang Y and Kim HS. Six-month versus 12-month dual antiplatelet therapy after implantation of drug-eluting stents: the Efficacy of Xience/Promus Versus Cypher to Reduce Late Loss After Stenting (EXCELLENT) randomized, multicenter study. Circulation. 2012;125:505-13.

15. Kedhi E, Fabris E, van der Ent M, Buszman P, von Birgelen C, Roolvink V, Zurakowski A, Schotborgh CE, Hoorntje JCA, Eek CH, Cook S, Togni M,

Meuwissen M, van Royen N, van Vliet R, Wedel H, Delewi R and Zijlstra F. Six months versus 12 months dual antiplatelet therapy after drug-eluting stent implantation in ST-elevation myocardial infarction (DAPT-STEMI): randomised, multicentre, non-inferiority trial. Bmj. 2018;363:k3793.

16. Hahn JY, Song YB, Oh JH, Cho DK, Lee JB, Doh JH, Kim SH, Jeong JO, Bae JH, Kim BO, Cho JH, Suh IW, Kim DI, Park HK, Park JS, Choi WG, Lee WS, Kim J, Choi KH, Park TK, Lee JM, Yang JH, Choi JH, Choi SH and Gwon HC. 6-month versus 12-month or longer dual antiplatelet therapy after percutaneous coronary intervention in patients with acute coronary syndrome (SMART-DATE): a randomised, open-label, non-inferiority trial. Lancet. 2018;391:1274-1284.

17. Nakamura M, Iijima R, Ako J, Shinke T, Okada H, Ito Y, Ando K, Anzai H, Tanaka H, Ueda Y, Takiuchi S, Nishida Y, Ohira H, Kawaguchi K, Kadotani M, Niinuma H, Omiya K, Morita T, Zen K, Yasaka Y, Inoue K, Ishiwata S, Ochiai M, Hamasaki T and Yokoi H. Dual Antiplatelet Therapy for 6 Versus 18 Months After Biodegradable Polymer Drug-Eluting Stent Implantation. JACC Cardiovasc Interv. 2017;10:1189-1198.

18. Didier R, Morice MC, Barragan P, Noryani AAL, Noor HA, Majwal T, Hovasse T, Castellant P, Schneeberger M, Maillard L, Bressolette E, Wojcik J, Delarche N, Blanchard D, Jouve B, Ormezzano O, Paganelli F, Levy G, Sainsous J, Carrie D, Furber A, Berlan J, Darremont O, Le Breton H, Lyuycx-Bore A, Gommeaux A, Cassat C, Kermarrec A, Cazaux P, Druelles P, Dauphin R, Armengaud J, Dupouy P, Champagnac D, Ohlmann P, Ben Amer H, Kiss RG, Ungi I and Gilard M. 6- Versus 24-Month Dual Antiplatelet Therapy After Implantation of Drug-Eluting Stents in Patients Nonresistant to Aspirin: Final Results of the ITALIC Trial

(Is There a Life for DES After Discontinuation of Clopidogrel). JACC Cardiovasc Interv. 2017;10:1202-1210.

19. Valgimigli M, Campo G, Monti M, Vranckx P, Percoco G, Tumscitz C, Castriota F, Colombo F, Tebaldi M, Fucà G, Kubbajeh M, Cangiano E, Minarelli M, Scalone A, Cavazza C, Frangione A, Borghesi M, Marchesini J, Parrinello G and Ferrari R. Short- versus long-term duration of dual-antiplatelet therapy after coronary stenting: a randomized multicenter trial. Circulation. 2012;125:2015-26.

20. Collet JP, Silvain J, Barthélémy O, Rangé G, Cayla G, Van Belle E, Cuisset T, Elhadad S, Schiele F, Lhoest N, Ohlmann P, Carrié D, Rousseau H, Aubry P, Monségu J, Sabouret P, O'Connor SA, Abtan J, Kerneis M, Saint-Etienne C, Beygui F, Vicaut E and Montalescot G. Dual-antiplatelet treatment beyond 1 year after drug-eluting stent implantation (ARCTIC-Interruption): a randomised trial. Lancet. 2014;384:1577-85.

21. Mauri L, Kereiakes DJ, Yeh RW, Driscoll-Shempp P, Cutlip DE, Steg PG, Normand SL, Braunwald E, Wiviott SD, Cohen DJ, Holmes DR, Jr., Krucoff MW, Hermiller J, Dauerman HL, Simon DI, Kandzari DE, Garratt KN, Lee DP, Pow TK, Ver Lee P, Rinaldi MJ and Massaro JM. Twelve or 30 months of dual antiplatelet therapy after drug-eluting stents. N Engl J Med. 2014;371:2155-66.

22. Lee CW, Ahn JM, Park DW, Kang SJ, Lee SW, Kim YH, Park SW, Han S, Lee SG, Seong IW, Rha SW, Jeong MH, Lim DS, Yoon JH, Hur SH, Choi YS, Yang JY, Lee NH, Kim HS, Lee BK, Kim KS, Lee SU, Chae JK, Cheong SS, Suh IW, Park HS, Nah DY, Jeon DS, Seung KB, Lee K, Jang JS and Park SJ.

Optimal duration of dual antiplatelet therapy after drug-eluting stent implantation: a randomized, controlled trial. Circulation. 2014;129:304-12.

23. Park SJ, Park DW, Kim YH, Kang SJ, Lee SW, Lee CW, Han KH, Park SW, Yun SC, Lee SG, Rha SW, Seong IW, Jeong MH, Hur SH, Lee NH, Yoon J, Yang JY, Lee BK, Choi YJ, Chung WS, Lim DS, Cheong SS, Kim KS, Chae JK, Nah DY, Jeon DS, Seung KB, Jang JS, Park HS and Lee K. Duration of dual antiplatelet therapy after implantation of drug-eluting stents. N Engl J Med. 2010;362:1374-82.

24. Helft G, Steg PG, Le Feuvre C, Georges JL, Carrie D, Dreyfus X, Furber A, Leclercq F, Eltchaninoff H, Falquier JF, Henry P, Cattan S, Sebagh L, Michel PL, Tuambilangana A, Hammoudi N, Boccara F, Cayla G, Douard H, Diallo A, Berman E, Komajda M, Metzger JP and Vicaut E. Stopping or continuing clopidogrel 12 months after drug-eluting stent placement: the OPTIDUAL randomized trial. Eur Heart J. 2016;37:365-74.
